# Dietary polyphenol intake, body composition and components of metabolic syndrome in a sample overweight and obese adults: a cross-sectional study

**DOI:** 10.1186/s12902-023-01507-y

**Published:** 2023-11-27

**Authors:** Shadia Hamoud Alshahrani, Zaid Al-Attar, Hamid Mahmood Abdullah Daabo, Najim Z. Alshahrani, Sarmad Ghazi Al-Shawi, Edgar Froilan Damián Núñez, Beneen M. Hussien, Ahmed Hjazi, Zahraa F. Hassan

**Affiliations:** 1 Medical Surgical Nursing Department, King Khalid University, Almahala, khamis mushate, Saudi Arabia; 2 Al-Kindy College of Medicine, HLA unit, University of Baghdad, Baghdad, Iraq; 3 Dental Technology Department, Technical College of Duhok, Duhok Polytechnic University, Duhok, Iraq; 4 Department of Family and Community Medicine, Faculty of Medicine, University of Jeddah, Jeddah, 21589 Saudi Arabia; 5 Food Science Department-Agriculture College, Basrah University, Basrah, Iraq; 6 Universidad Nacional Mayor de San Marcos, Lima, Perú; 7 Medical Laboratory Technology Department, College of Medical Technology, The Islamic University, Najaf, Iraq; 8 Department of Medical Laboratory Sciences, College of Applied Medical Sciences, Prince Sattam bin Abdulaziz University, Al-Kharj, 11942 Saudi Arabia; 9 College of Dentistry, Al-Ayen University, Thi-Qar, Iraq

**Keywords:** Dietary polyphenol intake, Metabolic parameters, Overweight, Obesity, Glycemic markers

## Abstract

**Background:**

The health benefits of dietary polyphenol intake (DPI) including improved lipid profiles, blood pressure, insulin resistance, and reduced systemic inflammation has revealed previously. However, the results of numerous studies are not consistent and it seems that these health effects are attributed to some of DPI. In the current research, we evaluated the health benefits of DPI on metabolic markers and glycemic markers among overweight and obese individuals.

**Methods:**

In this cross-sectional study, 487 individuals with overweight and obesity were participated. Dietary intake was assessed by a Food Frequency Questionnaire (FFQ) and the amount of dietary polyphenol intake were calculated based on the information derived from Phenol-Explorer database (www.phenolexplorer.eu/contents). Bioelectrical impedance analysis (BIA) was used to measure body composition. Systolic and diastolic blood pressures were measured by sphygmomanometer. Biochemical assays including fasting blood sugar, insulin and serum lipids’ concentrations were measured by enzymatic methods.

**Results:**

According to our results, males were more likely to be at the highest tertile of DPI (*P* = 0.04). Also, those at the highest tertile of DPI had higher fat free mass and physical activity level (*P* < 0.05). Lower TG level in highest tertile of DPI in crude model was also observed, but, it lost its significant threshold after adjustment for confounders. Subjects at the second tertile of DPI were more likely to have lower systolic blood pressure in the sex and age adjusted model [OR = 0.970; CI = 0.940-1.000; *P* = 0.049]. For other biochemical variables, no significant association was observed.

**Conclusion:**

In the current study, total dietary polyphenol intake was associated with lower SBP among overweight and obese individuals. Further studies are warranted to better elucidate the observed results.

**Supplementary Information:**

The online version contains supplementary material available at 10.1186/s12902-023-01507-y.

## Introduction

Obesity is a major health problem and is considered as a chronic low grade inflammatory status [1, 2]. Obesity is associated with numerous comorbidities including type two diabetes mellitus (T_2_DM), metabolic syndrome (MetS), cardiovascular disorders (CVD), respiratory dysfunction, mental problems and some types of cancers [3–8]. MetS is closely associated with obesity status and its prevalence is increasing obesity prevalence as “Obesity Pandemic” [9]. Metabolic syndrome is a combination of numerous abnormalities that triggers the progression of CVD and T_2_DM and according to the definition of National Cholesterol Education Program Adult Treatment Panel (NCEP-ATP) III, it is defined as a combination of central obesity, hypertension, hypertriglyceridemia and higher amount of low-density lipoprotein cholesterol levels (LDL-c), and lower levels of high-density lipoprotein cholesterol (HDL-c) [10]. There are numerous changeable and unchangeable risk factors that are potent predictors of obesity and metabolic syndrome; unchangeable risk factors includes age, gender, genetics, race, etc. [11]. while, changeable and modifiable risk factors are dietary style, physical activity and psychological modifications [12, 13]. Healthy diet, play an important role in human health and unhealthy dietary behaviors and dietary habits are potent triggers of chronic disease status [14, 15]; among dietary factors, the significant and amazing effects of dietary polyphenols intake (DPI) attracted great attention in recent years because of their anti-oxidant and anti-inflammatory properties [16, 17]. Polyphenols are a member of natural dietary phytochemicals that also include other ingredients like terpenoids, organosulfurs and phytosterols [18]; polyphenols, with several hydroxyl groups on an aromatic ring, modulate oxidative response, inflammatory and cell proliferative processes that are also responsible for initiation of several chronic diseases including T_2_DM, metabolic syndrome, obesity, cardiovascular diseases (CVD) and cancers [19, 20]. Polyphenolic compounds exist in almost all medicinal and edible herbs. The richest dietary sources of polyphenolic compounds are fruits, seeds, vegetables and spices. Also, certain beverages like coffee, tea, and wine contribute significantly to the daily intake of phenolic compounds [21]. Phenolic compounds are classified into four major groups: simple phenols (e.g. phenolic acid), lignans, stilbenes, and polyphenols (e.g. flavonoids) [22]. Due to their chemical structure, polyphenols exert numerous health effects against chronic disease and there are numerous epidemiological evidence in this filed; in a 6-year cohort study by Adriouch S et al., higher intake of dietary polyphenols (e.g. more than 915 mg/d) including flavanones and lignans were associated with reduced body mass index (BMI) and waist circumference (WC) over six years follow-up among middle-aged adults [23]. It has been suggested that anti-adipogenic activities of polyphenols are applied through the mechanistic target of rapamycin (mTOR) signaling pathway that is involved in the pathogenesis of numerous other comorbidities including diabetes, metabolic syndrome and CVD [24]. In another cross-sectional study by Yeon JY et al. [25], mean flavones and flavonols intakes of 37.34 mg/ d among men and 46.78 among women were inversely associated with insulin resistance among 4,186 Korean male subjects. In another study in Polish adults, significant associations were observed between high DPI (e.g. more than 3092 mg/d in men and 2904 mg/d in women) and lower fasting blood glucose, blood pressure, and HDL-c [26]. Dietary behaviors and habits have different geographical distributions and vary from one region to another. Because of limited number of studies regarding the association between DPI intake and cardio-metabolic risk factors in Iraq, in the current study, we aimed to investigate the association between DPI and anthropometric and metabolic risk factors in a sample of obese population in Saudi Arabia.

## Materials and methods

### Study design and study population

In the current cross-sectional study, 487 random samples of overweight/obese participants aged between 20 and 50 years old were invited by public announcements in Abha, Saudi Arabia. Overweight and obesity were defined according to the World Health Organization (WHO) Criteria of having body mass index greater than or equal to 25 and 30 kg/m^2^ respectively [27, 28]. The exclusion criteria were pregnancy, lactation, post menopause, any history of bariatric surgeries, and any history of chronic disease like CVD, T_2_DM or cancer, liver or kidney problems, and use of medication or supplements that might affect weight status. All participants read and signed an informed consent form, also the Princess Nourah bint Abdulrahman University Ethics Committee (Identifier: PNURSP2023R259) has approved the study protocol.

### Socio-demographic, anthropometric and physical activity assessment

Demographic information including educational attainment, marital status, household size and occupation were gathered through questionnaires and interviews. Weight and height were measured to the nearest 0.1 in kg and 0.1 cm, respectively using Seca scale (Seca co., Hamburg, Germany). WC was measured to the nearest 0.1 cm at the midpoint between the lowest rib margin and the iliac crest. Hip circumference was also measured with a non-stretchable tape as the distance around the largest part of the hips. Physical activity was assessed by international physical activity questionnaire (IPAQ) [29–31]. The bioelectrical impedance analysis (BIA) method was employed by Tanita, BC-418 MA (Tanita Corporation, Tokyo, Japan) providing information about fat mass (FM) and fat free mass (FFM).

### Blood pressure and biochemical assays

Blood pressure including systolic blood pressure (SBP) and diastolic blood pressure (DBP) was measured with a trained physician used a standard mercury sphygmomanometer (OMRON M6). In order to perform biochemical assays, 10 ml of blood samples were obtained and sera were extracted. Individuals’ serum specimens were stored frozen until assay. Blood lipids including serum total cholesterol (TC), high-density lipoprotein cholesterol (HDL-C) and triglycerides (TG) and fasting serum glucose (FSG) were analyzed by enzymatic assays using the Roche/Hitachi Cobas 6000 Auto- Analyzer (Roche Diagnostics, 9115 Hague Road, Indianapolis, IN, USA). The principal of this analyzer for TC, is conversion of esterified cholesterol to cholesterol by cholesterol esterase. The cholesterol is then converted to cholest-4-en-3-one and hydrogen peroxide by cholesterol oxidase. The hydrogen peroxide then reacts with 4-aminophenazone in the presence of peroxidase to produce a colored product that is measured at 505 nm wavelength. For TG, the corresponding method is hydrolyzing TG by lipase, conversion to glycerol and fatty acids, phosphorylation of glycerol to glycerol phosphate with glycerol kinase and its oxidation with glycerol phosphokinase to dihydroxy acetone and H_2_O_2_. For HDL, The automated HDL assay uses detergents, cholesterol esterase, cholesterol oxidase and peroxidase to form a colored pigment that is measured enzymatically [32]. Serum insulin levels were measured by commercial kits (Bioassay Technology Laboratory, Shanghai Korean Biotech, Shanghai City, China). The Friedewald Eq. [33] was used to calculate the amount of low-density lipoprotein cholesterol (LDL-C). The Homeostasis Model Assessment of Insulin Resistance (HOMA-IR) was calculated by following formula: blood insulin (IU/ml)/22.5 blood glucose (mmol/l), and the Quantitative Insulin Sensitivity Check Index (QUICKI) was estimated as: 1/ [log fasting insulin level (U/mL) + log fasting glucose level (mmol/L) [34].

### Dietary data and DPI calculation

Dietray intake was evaluated using a validated, semi-quantitative food frequency questionnaire (FFQ) with 140 food items, with acceptable validity and reliability, that was adapted for the Saudi’s general population [35]. The Saudi’s household manual’s recommendations for cooking yields and portion sizes were used to gather information about participants’ food intake and beverage consumption. The amounts then were converted to grams. We used data provided in Phenol-Explorer database (www.phenolexplorer.eu/contents) to calculate the DPI [36]. The total polyphenol content was measured using the Folin Ciocalteu (F-C) assay as sum of four main subgroups (including flavonoids, phenolic acids, stilbenes, lignans, and other polyphenols). The F–C assay provides the reducing capacity of a sample, expressed in terms of phenolic content measured as total phenolic content with HPLC-UV or quantification by HPLC‐MS/MS. The F-C commercial reagents are available [37, 38]. The polyphenol content of all of the food groups including nuts, fruits and its products, fats and oils, vegetables, legumes, beverages, jams, milk and products, cereals and cereal products, dairy and spices were calculated and imported into the DPI. Supplementary Table 1, provides the polyphenol contents of different food groups that are used in the current study. Also, polyphenolic content of food groups with summation of all of the related food items ranging from highest to the lowest polyphenolic content is provided in Table 1.

**Table 1:** Total polyphenol content of food groups studied in the current research

Food group/ item ^a^	Polyphenol content (mg/g)
Nuts	1738.71
Vegetables	1569.25
Fruits and its products	1343.18
Beverages	1060.35
Fat and oils	1056.44
Cereals and its products	512.81
Legumes	391.39
Chocolate (dark, milk)	280.72
Jams	175.76
Meats	102.35
Milk (chocolate)	19.22

^a^Food groups are ranked from the highest to the lowest amount of polyphenols

### Statistical analysis

All data were analyzed using SPSS (version 21.0; SPSS Inc, Chicago IL). The normality of the data was investigated using histogram charts and the Kolmogorov-Smirnov test. Normal distributes quantitative data presented as a mean (SD), and for qualitative data as a frequency (%).The Chi-square test and one-way analysis of variance (ANOVA) were used to compare differences in discrete and continuous variables among different tertiles of DPI, respectively. In three multivariable-adjusted models, the association between the DPI tertiles and biochemical variables was examined using multinomial logistic regression to estimate odds ratios (ORs) and 95% confidence intervals (CIs) for the presence of cardiometabolic risk factors among the DPI tertiles.

## Results

Table 2, presents the comparison of demographic variables by different dietary DPI tertiles. Those at the highest tertile of DPI were more likely to be male subjects (*P* = 0.04), to have higher fat free mass (*P* = 0.03) and to have higher level of physical activity (*P* = 0.03). Lower TG level in highest tertile of DPI in crude model of ANOVA was also observed (*P* = 0.03). However, in ANCOVA model after adjustment for confounders, this significance level was lost. Also, those at the highest level of DPI had higher intake of dietary fruits, vegetables, fiber, saturated fatty acid (SFA), monounsaturated fatty acid (MUFA), polyunsaturated fatty acid (PUFA) in both crude and age, gender, BMI, PA and energy intake-adjusted models (Table 3). In evaluation of the odds of biochemical variables in different DPI tertiles (Table 4), those at the second tertile of DPI were more likely to have lower SBP [OR = 0.970; CI = 0.940-1.000; *P* = 0.049] only in age and sex adjusted model. No other significant association was observed for other biochemical variables and in the third tertile of DPI.

**Table 2:** General demographic characteristics of study participants by tertiles of DPI

Variable	Tertiles of DPI							
	1st (*N*=162)		2nd (*N*=163)		3rd (*N*=162)		P* value	P*** value
	596.16-643.43 (mg/d)		1103.12-1152.88 (mg/d)		1944.41-2305.54 (mg/d)			
	**Mean**	**SD**	**Mean**	**SD**	**Mean**	**SD**		
Age (y)	39.65	9.21	40.76	8.74	41.53	9.55	0.30	-
Education [n (%) ≤ 12 y)]	29	25.7	21	18.2	30	25.8	0.72**	-
Marital status [n (%) Single]	13	11.5	14	12.3	18	15.9	0.99**	-
Gender [n (%) Male]	58	51.3	65	57	73	64.5	0.044**	-
Weight (kg)	91.49	14.11	91.13	14.13	93.70	15.22	0.35	-
Height (cm)	167.46	9.58	168.3684	10.11	168.05	9.99	0.78	-
BMI (kg/m^2^)	32.70	4.99	32.17	4.65	33.10	4.87	0.35	-
WC (cm)	105.93	9.44	106.56	9.69	107.65	9.83	0.40	-
FM (%)	35.48	7.83	32.58	10.09	33.19	9.39	0.17	-
FFM (%)	59.04	11.36	63.31	12.81	64.58	12.42	**0.03**	-
WHR	0.92	0.09	0.93	0.07	0.95	0.07	0.16	-
BMR (Kcal)	7474.01	1507.42	8032.14	1487.34	8089.51	1776.50	0.06	-
PA (met. min/week)	1339.94	2237.17	2769.06	3079.09	2468.53	3963.13	**0.03**	-
SBP (mmHg)	122.19	15.08	122.27	17.18	124.11	17.05	0.61	0.93
DBP (mmHg)	80.46	11.53	82.75	12.54	81.80	11.01	0.34	0.26
FBS (mg/dl)	93.54	15.73	91.27	14.86	93.46	25.70	0.61	0.76
TC (mg/dl)	193.31	37.92	195.69	40.15	186.37	30.98	0.14	0.24
TG (mg/dl)	147.92	94.24	168.66	113.24	136.20	62.55	**0.03**	0.92
HDL (mg/dl)	43.87	10.15	43.25	9.70	43.39	8.67	0.88	0.93
LDL (mg/dl)	126.65	33.51	124.66	34.75	119.63	26.78	0.24	0.15
Insulin (mIU/l)	16.37	10.23	17.21	17.93	14.54	11.06	0.44	0.50
HOMA-IR	3.88	2.63	3.92	4.06	3.41	2.85	0.53	0.57
QUICKI	0.33	0.03	0.33	0.04	0.33	0.03	0.39	0.41

All data are mean (± SD) except for education, marital status and gender, that are presented as the number and percent

*BMI* Body mass index, *WC* Waist Circumference, *FM* Fat Mass, *FFM* Fat Free Mass, *WHR* Waist-to-hip ratio, *BMR* Basal Metabolic Rate, *PA* Physical Activity, *SES* Socio-economic status, *SBP* Systolic Blood Pressure, *DBP* Diastolic Blood Pressure, *TC* Total Cholesterol, *TG* Triglyceride, *HDL-C* High Density Lipoprotein Cholesterol, *LDL-C* Low Density Lipoprotein Cholesterol, *HOMA-IR* Homeostatic Model Assessment for Insulin Resistance, *QUICKI* Quantitative Insulin sensitivity Check Index

P* values derived from One-Way ANOVA with Tukey’s post-hoc comparisons

** *P* values derived from chi-squared test

P*** values derived from One-Way ANOVA with Tukey’s post-hoc comparisons after adjustment for confounders (age, gender, BMI, PA and energy intake)

**Table 3:** Food groups intake of study participants by tertiles of DPI

Variable	Tertiles of DPI										
	1st (*N*=162)			2nd (*N*=163)			3rd (*N*=162)			P* value	P** value
	596.16-643.43 (mg/d)			1103.12-1152.88 (mg/d)			1944.41-2305.54 (mg/d)				
	**N**	**Mean**	**SD**	**N**	**Mean**	**SD**	**N**	**Mean**	**SD**		
Fruits (g/d)	^162^	3.11	1.79	163	3.91	2.01	162	5.43	4.32	< 0.001	**< 0.001**
Vegetables (g/d)	^162^	3.00	1.82	163	3.79	1.53	162	4.78	2.80	< 0.001	**< 0.001**
Fiber (g/d)	^162^	58.47	31.08	163	72.20	44.04	162	85.81	50.82	< 0.001	**0.004**
Energy (kcal/d)	^162^	2527.97	868.93	163	2924.89	872.48	162	3608.52	1234.29	< 0.001	**< 0.001**
CHO (%)	^162^	57.85	7.12	163	57.40	6.89	162	58.53	6.78	0.66	0.705
Protein (%)	^162^	13.22	1.67	163	13.29	2.09	162	12.57	2.11	0.07	**0.026**
Fat (%)	^162^	31.46	7.20	163	31.83	7.33	162	31.75	6.38	0.95	0.706
Cholesterol (mg/d)	^162^	261.29	252.73	163	276.54	136.74	162	352.90	180.17	0.01	0.244
SFA (g/d)	^162^	24.71	11.30	163	28.00	11.97	162	35.34	18.66	< 0.001	**< 0.001**
MUFA (g/d)	^162^	28.16	14.21	163	31.75	14.34	162	39.99	19.01	< 0.001	**< 0.001**
PUFA (g/d)	^162^	18.74	10.04	163	21.32	11.55	162	27.81	15.90	< 0.001	**< 0.001**

*CHO* Carbohydrate, *SFA* Saturated fatty acids, *MUFA* Mono-unsaturated fatty acids, *PUFA* polyunsaturated fatty acids. All data are mean (± SD)

P* values derived from unadjusted ANCOVA. P** values derived from ANCOVA after adjustment for confounders (age, gender, BMI, PA and energy intake)

**Table 4:** Biochemical variables of study participants by tertiels of DPI

Variable		Tertiels of DPI				
		1st (*N*=162)	2nd (*N*=163)		3rd (*N*=162)	
		596.16-643.43 (mg/d)	1103.12-1152.88 (mg/d)		1944.41-2305.54 (mg/d)	
			OR(CI)	*P*-value	OR(CI)	*P*-value
SBP (mmHg)	Model I	**1** **REF**	0.974(0.945–1.004)	0.09	1.004 (0.976–1.034)	0.77
	Model II		0.970 (0.940-1.000)	**0.049**	0.999 (0.970–1.029)	0.95
	Model III		0.983 (0.904–1.070)	0.70	1.005 (0.919–1.099)	0.91
DBP (mmHg)	Model I	**1** **REF**	1.042 (0.999–1.086)	0.06	1.017 (0.977–1.059)	0.40
	Model II		1.047 (0.989–1.092)	0.09	1.022 (0.980–1.065)	0.31
	Model III		1.036 (0.939–1.144)	0.48	1.016 (0.913–1.129)	0.77
FBS (mg/dl)	Model I	**1** **REF**	0.991 (0.958–1.024)	0.58	1.011 (0.979–1.043)	0.51
	Model II		0.985 (0.952–1.020)	0.40	1.003 (0.971–1.037)	0.85
	Model III		0.985 (0.907–1.071)	0.73	1.012 (0.932–1.099)	0.78
TC (mg/dl)	Model I	**1** **REF**	0.997 (0.975–1.019)	0.77	1.013 (0.978–1.049)	0.48
	Model II		0.996 (0.974–1.018)	0.71	1.014 (0.977–1.052)	0.46
	Model III		0.995 (0.973–1.017)	0.63	0.980 (0.957–1.004)	0.11
TG (mg/dl)	Model I	**1** **REF**	1.005 (0.999–1.010)	0.10	0.997 (0.990–1.005)	0.46
	Model II		1.004 (0.999–1.010)	0.12	0.996 (0.989–1.004)	0.38
	Model III		1.005 (0.993–1.017)	0.40	1.008 (0.996–1.021)	0.18
HDL (mg/dl)	Model I	**1** **REF**	1.001 (0.963–1.041)	0.94	0.980 (0.934–1.028)	0.40
	Model II		1.011 (0.971–1.053)	0.59	0.991 (0.943–1.042)	0.73
	Model III		0.942 (0.834–1.064)	0.33	1.003 (0.881–1.141)	0.97
LDL (mg/dl)	Model I	**1** **REF**	1.004 (0.981–1.028)	0.73	0.977 (0.942–1.014)	0.22
	Model II		1.005 (0.982–1.029)	0.67	0.976 (0.940–1.014)	0.21
	Model III		1.051 (0.988–1.29)	0.76	0.99 (1.01–1.032)	0.56
Insulin (mIU/l)	Model I	**1** **REF**	1.010 (0.871–1.171)	0.89	1.043 (0.900-1.208)	0.58
	Model II		0.990 (0.850–1.153)	0.90	1.017 (0.874–1.184)	0.82
	Model III		0.967 (0.563–1.663)	0.90	1.124 (0.688–1.836)	0.64
HOMA-IR	Model I	**1** **REF**	1.020 (0.549–1.894)	0.95	0.840 (0.461–1.531)	0.57
	Model II		1.113 (0.592–2.090)	0.74	0.934 (0.505–1.728)	0.83
	Model III		1.048 (0.155–7.104)	0.96	1.017 (0.251–4.121)	0.98
QUICKI	Model I	**1** **REF**	1.005 (0.999–1.010)	0.51	1.009 (0.998–1.091)	0.29
	Model II		0.990 (1.09–1.021)	0.61	1.008 (0.996–1.021)	0.39
	Model III		1.090 (1.00-1.010)	0.97	0.980 (0.934–1.028)	0.16

The multivariate multinomial logistic regression was used for estimation of ORs and confidence interval (CI). Model I: crude, Model II: adjusted for age and sex, Model III: adjusted for age, BMI, sex, physical activity, and energy intake

Systolic Blood Pressure; *DBP *Diastolic Blood Pressure, *TC* Total Cholesterol, *TG* Triglyceride, *HDL-C* High Density Lipoprotein Cholesterol, *LDL-C* Low Density Lipoprotein Cholesterol, *HOMA-IR* Homeostatic Model Assessment for Insulin Resistance, *QUICKI* Quantitative Insulin sensitivity Check Index, *OR* odds ratio, *CI* confidence interval

## Discussion

**Fig. 1:**
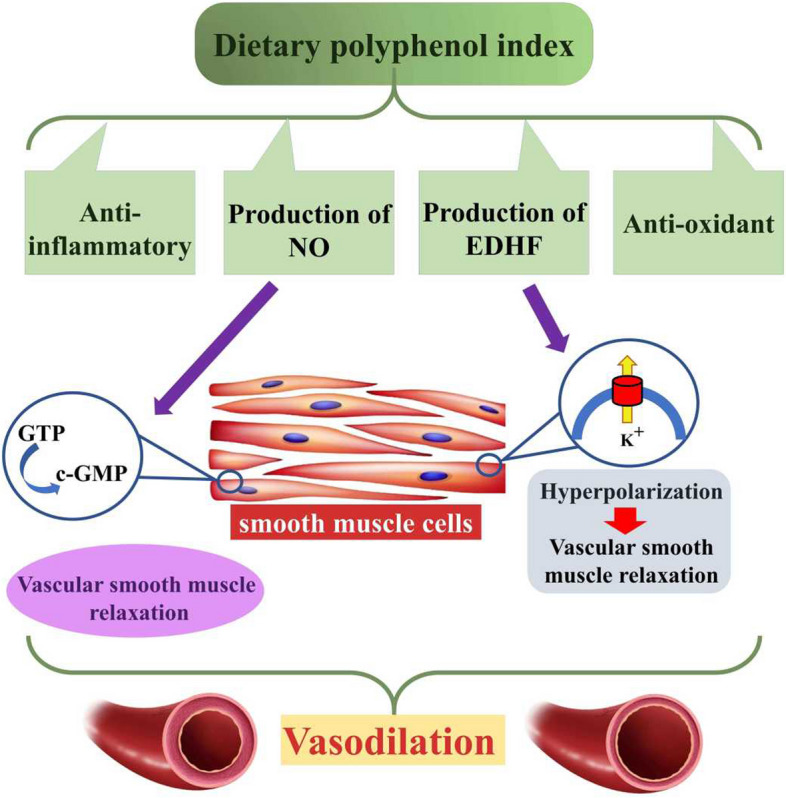
The mechanism of the effect of DPI on hypertension, polyphenols with anti-inflammatory and antioxidant properties are directly effective in reducing blood pressure [52], meanwhile, polyphenols have vasodilation effects by producing NO and converting GTP to c-GMP [53], as well as producing EDHF and hyperpolarization, followed by relaxation of vascular smooth muscles [46]. DPI, dietary polyphenol intake; NO, nitric oxide; EDHF, endothelium-derived hyperpolarizing factor; GTP, guanosine triphosphate; cGMP, cyclic guanosine monophosphate

In the current study, for the first time we evaluated the association between dietary polyphenol intake obtained from a wide varieties of food groups with metabolic parameters among overweight and obese adults in Saudi Arabia. We observed a lower odds of SBP at those in the second tertile of DPI versus first tertile in age-and sex adjusted model. A significantly lower TG also was observed in the highest tertiles of DPI versus lowest (*P* = 0.03), although it was lost after adjustment for confounders. In the a cross-sectional study by Aali Y et al. [39], dietary polyphenol intake greater than 2731.68 mg /d obtained from a limited number of food groups was associated with lower cardio-metabolic risk factors among obese adults [39]. Same as our results, in a population based study in Brazil [40], an inverse and linear association was observed between the hypertension and total dietary polyphenol intake in the middle tertile of DPI. The median intake of polyphenols was 360.6 mg/d ranging from 233.0 to 478.6 mg/d. Although in the mentioned study, in the separate analysis of polyphenols, some of their classes were associated with reduced blood pressure in highest tertile. But, we did not reported the isolate effects of dietary polyphenols because our study, mostly focuses on the usual dietary intakes and in a real diet, we are consuming all of these ingredients altogether not isolate. The results of studies regarding the association between DPI and blood pressure are inconsistent. For example, in the study by Jennings A et al. [41], that was conducted among 1898 women, aged from 18 to 75 years old in UK, among the various groups of compounds of polyphenols with total mean intake of 1149 ± 661 mg/d, only anthocyanins subclass with mean intake of 17.7 ± 14.9 mg/d were negatively associated with lower systolic blood pressure. In the Healthy Lifestyle in Europe by Nutrition in Adolescence (HELENA) study [42], total polyphenol intake was not related with blood pressure. Total polyphenol intake in HELENA study ranged from mean of 51.8 ± 22.4 mg/1000 kcal in the lowest quartile to mean of 458.9 ± 281.8 mg/1000 kcal in the highest quartile. HELENA was a cross-sectional HELENA study, involving 657 adolescents with 12.5–17.5 mean age, while 54% of participants were girls and 14.8% were overweight. The fasting blood sample and polyphenol intake data in HELENA study were obtained from two non-consecutive 24-hour recalls matched with the Phenol-Explorer database. MetS was defined via the pediatric American Heart Association definition. In one other cross-sectional study with 2618 adults, aged 19 to 84 years old, among total polyphenols with median dietary intake of 1013 mg/d, only flavonoid intake with 549 mg/d was inversely associated with lower blood pressure [43]. While, in the study by Grosso G et al. [44], total and all of the subclasses of DPI were inversely associated with risk of metabolic syndrome. In that study dietary total polyphenol intake was between 1060 and 1091 mg/d in men and 1109–1123 mg/d among women. The possible antihypertensive mechanisms of DPI is toward their anti-inflammatory and antioxidant effects; it is suggested that the polyphenols can exert vaso-protective effects via production of nitric oxide (NO) and endothelium-derived hyperpolarizing factor (EDHF), promote vasodilatation, reduce platelet aggregation, and improved vascular smooth muscle function, via reducing the excessive vascular oxidative stress [45, 46]. In the present study, the total polyphenol intake in the highest tertile was not significantly associated with lower odds of hypertension that was similar to the study of Miranda AM et al. [40]. It can be explained by the difference in the food preferences and dietary habits of our populations. As explained before, the dietary pattern of our population can affect the intake of different subclasses of polyphenols. Some of the subclasses of polyphenols are revealed to even increase blood pressure like coffee because of its caffeine [47]. In our study, the lower TG in highest versus lowest tertile of DPI was lost after adjustment for confounders. Although this inverse trend for serum lipids was also observed for TC and LDL in a non-significant threshold. The anti-hyperlipidemic effects of DPI are also reported in previous studies; in the study by Castro-Barquero S. et al. [48], total polyphenol intake was associated with increased HDL cholesterol but not with other lipids. Also, in isolate analysis of polyphenols, dietary intake of stilbene and lignan were inversely associated with serum TG. Reduced oxidative damage to lipids [49], protection against peroxidative damage-related lipid degradation and up-regulation of gene expression related to lipid metabolism [50] are proposed mechanisms of anti-hyperlipidemic actions of polyphenols. Although, because of great between-studies heterogeneities, further studies are warranted [51] to illustrate the real causality. Some of mechanistic pathways are summarized in Fig. 1. The current cross-sectional study has some strengths including relatively appropriate sample size, the use of the Phenol-Explorer as the most comprehensive food composition database on dietary polyphenols [36] and using a validated FFQ for Saudi population. However, the limitations of the current study should also be addressed; first, its cross-sectional design which limits the interpretation of causality. Second, the study was performed among overweight and obese individuals in Saudi Arabia and therefore, it cannot be generalized into other populations; third some of the factors related to foods’ polyphenol content like food bioavailability, variety and environmental conditions are not studied.

In conclusion, in the current cross-sectional study among overweight and obese adults, total dietary polyphenol intake in the second tertile, was associated with lower SBP in age and sex adjusted model. Also, a lower TG in unadjusted model of ANOVA was observed in highest versus lowest tertile of total dietary polyphenol intake.

The results of the current study have some implications for future researches, first of all, further studies among other populations with different disease status and preferably, with interventional or longitudinal designs are warranted to better elucidate the causality. Also, it is suggested that the association of dietary polyphenol intake with other metabolic factors including inflammatory parameters and other more specific markers of glucose homeostasis like HbA1C also be investigated.

### Supplementary Information

**Additional file 1.**

## Data Availability

The datasets used and/or analyzed during the current study available from the corresponding author on reasonable request.

## Abbreviations

NCEP-ATP: National Cholesterol Education Program Adult Treatment Panel
LDL-c: Low-density lipoprotein cholesterol
HDL-c: High-density lipoprotein cholesterol
DPI: Dietary polyphenols intake
CVD: Cardiovascular diseases
mTOR: Mechanistic target of rapamycin
BMI: Body mass index
WC: Waist circumference
WHO: World Health Organization
T_2_DM: Type two diabetes mellitus
MetS: Metabolic syndrome
IPAQ: International physical activity questionnaire
TG: Triglycerides
FSG: Fasting serum glucose
HOMA-IR: Homeostasis Model Assessment of Insulin Resistance
QUICKI: Quantitative Insulin Sensitivity Check Index
FFQ: Food frequency questionnaire
ORs: Odds ratios
SBP: Systolic blood pressure
DBP: Diastolic blood pressure
HPLC-UV: High performance liquid chromatography ultra-violet
HPLC-MS/MS: Mass spectrometry
HELENA: Healthy Lifestyle in Europe by Nutrition in Adolescence
