# Supplementary material for: Dietary polyphenol intake, body composition and components of metabolic syndrome in a sample overweight and obese adults: a cross-sectional study
Source: BMC Endocr Disord. 2023 Nov 27;23:261. doi: 10.1186/s12902-023-01507-y (PMC10680328; doi:10.1186/s12902-023-01507-y)
Supplement: Supplementary file 1 — Additional file 1. [file 12902_2023_1507_MOESM1_ESM.docx]

| **Supplementary Table 1. Food items imported to extract dietary polyphenols** | | | | | | | | | | | | | | | | |
| --- | --- | --- | --- | --- | --- | --- | --- | --- | --- | --- | --- | --- | --- | --- | --- | --- |
| **Food items** | **Polyphenols contents** | | | | | | | **Total** | **Food items** | **Polyphenols contents** | | | | | | **Total** |
| **Nuts** | | | | | | | | | **Fruits and its products** | | | | | | | |
| Peanut | Flavonoids | | Isoflavonoids:0.47 | | | | | 0.47 | Cantaloupe | Lignans | Lignans:9.5 | | | | | 9.5 |
|  | Stilbene | | Stilbenes:0.08 | | | | | 0.08 |  |  |  |  |  |  |  |  |
|  | **Polyphenols total:0.55** | | | | | | | |  | **Polyphenols total:9.5** | | | | | | |
| Chestnut | Flavonoids | | Flavanols:3.02 | | | | | 3.02 | melon | Lignans | Lignans:1.12 | | | | | 1.12 |
|  | Phenolic acids | | Hydroxybenzoic acids:1215.22 | | | | | 1215.22 |  |  |  |  |  |  |  |  |
|  | **Polyphenols total:1218.24** | | | | | | | |  | **Polyphenols total :1.12** | | | | | | |
| Walnut | Phenolic acids | | Hydroxybenzoic acids:28.50 | | | | | 28.50 | Watermelon | Flavonoids | Flavones:1.84 | | | | | 1.84 |
|  |  |  |  |  |  |  |  |  |  | Lignans | Lignans:6.18 | | | | | 6.18 |
|  | **Polyphenols total :28.50** | | | | | | |  |  | **Polyphenols total:8.02** | | | | | | |
| Sunflower seed | Phenolic acids | Hydroxybenzoic acids:462.65 | | | | | | 462.65 | Pear (whole) | Flavonoids | Flavanols:4.05  Flavonols:0.77 | | | | | 4.82 |
|  |  |  |  |  |  |  |  |  |  | Phenolic acids | Hydroxybenzoic acids:0.54  Hydroxycinnamic acids:11.27 | | | | | 11.81 |
|  |  |  |  |  |  |  |  |  |  |  | Other polyphenols:0.05 | | | | | 0.05 |
|  | **Polyphenols total:462.65** | | | | | | | |  | **Polyphenols total:16.68** | | | | | | |
| Pistachio | Flavonoids | Flavanols:6.9 | | | | | | 6.9 | apricot | Flavonoids | Flavanols:8.57  Flavonols:0.95 | | | | | 9.52 |
|  |  |  |  |  |  |  |  |  |  | Phenolic acids | Hydroxycinnamic acids:10.04 | | | | | 10.04 |
|  | **Polyphenols total:6.9** | | | | | | | |  | **Polyphenols total:19.56** | | | | | | |
| Hazelnut | Flavonoids | Flavanols:5.7 | | | | | |  | Sweet cherry | Flavonoids | Anthocynins:171.42  Flavanols:15.07 | | | | | 186.49 |
|  |  |  |  |  |  |  |  |  |  | Phenolic acids | Hydroxycinnamic acids:85.81 | | | | | 85.81 |
|  | **Polyphenols total:5.7** | | | | | | |  |  | **Polyphenols total:272.30** | | | | | |  |
| Almond | Flavonoids | Flavanols:4.93  Flavanones:0.50  Flavonols:6.21 | | | | | | 11.64 | Apple | -- | | | | | |  |
|  | Phenolic acids | Hydroxybenzoic acids:4.53 | | | | | | 4.53 |  |  |  |  |  |  |  |  |
|  | **Polyphenols total:16.17** | | | | | | | |  |  |  |  |  |  |  |  |
| **Fats** | | | | | | | | | Peach | Flavonoids | Flavanols:2.33 | | | | | 2.33 |
|  |  |  |  |  |  |  |  |  |  | Phenolic acids | Hydroxycinnamic acids:24.85 | | | | | 24.85 |
|  |  |  |  |  |  |  |  |  |  | **Polyphenols total::27.80** | | | | | | |
| Olive oil; refined | flavonoids | | | Flavones:0.15 | | | | 0.15 | Nectarine ,whole | Flavonoids | Anthocynins:0.30  Flavanols:14.67  Flavonols:0.21 | | | | | 15.18 |
|  | Lignans | | | Lignans:3.16 | | | | 3.16 |  | Phenolic acids | Hydroxycinnamic acids:10.04 | | | | | 10.04 |
|  | Other polyphenols | | | Tyrosols:33.34  Other polyphenols:0.25 | | | | 33.59 |  | **Polyphenols total::25.22** | | | | | | |
|  | **Polyphenols total:36.9** | | | | | | | |  |  |  |  |  |  |  |  |
| Olive oils; virgin | Flavonoids | | | | Flavones:0.23 | | | 0.23 | Fresh fig | Lignans | Lignans:0.37 | | | | | 0.37 |
|  | Lignans | | | | Lignans:2.81 | | | 2.81 |  | **Polyphenols total::0.37** | | | | | | |
|  | Phenolic acids | | | | Hydroxybenzoic acids:12.54  Hydroxycinnamic acids:0.09  Hydroxyphenylacetic acids :10.55 | | | 23.18 |  |  |  |  |  |  |  |  |
|  | Other polyphenols | | | | Hydroxybenzaldehydes:0.02  Tyrosols: 69.49  Other polyphenols:  0.28 | | | 69.79 |  |  |  |  |  |  |  |  |
|  | **Polyphenols total :96.01** | | | | | | | |  |  |  |  |  |  |  |  |
| Rape seed oil | Phenolic acids | | | | | Hydroxybenzoic acids:0.68  Hydroxycinnamic acids:1.62 | | 2.3 | Dried figs | Lignans | Lignans:1.11 | | | | | 1.11 |
|  | Other polyphenols | | | | | Alkymethoxyphenols:14.43 | | 14.43 |  | **Polyphenols total :1.11** | | | | | | |
|  | **Polyphenols total:16.73** | | | | | | | |  |  |  |  |  |  |  |  |
| Sunflower seed oils | **Polyphenols total:1.**00 | | | | | | | | Grapes , black | Flavonoids | | | Anthocynins:72.1  Flavanols:14.03  Flavonols:3.08 | | | 89.21 |
|  |  |  |  |  |  |  |  |  |  | Phenolic acids | | | Hydroxycinnamic acids:1.69 | | | 1.69 |
|  |  |  |  |  |  |  |  |  |  | Stilbene | | | Stilbenes:6.53 | | | 6.53 |
|  |  |  |  |  |  |  |  |  |  | **Polyphenols total:97.43** | | | | | | |
| Olive, green , raw | Flavonoids | | | | | Flavones:0.56 | | 0.56 | Grapes, green | Flavonoids | | | Flavanols:3.78  Flavonols:2.49 | | | 6.27 |
|  | Phenolic acids | | | | | Hydroxybenzoic acids:13.84  Hydroxycinnamic acids:103.77  Hydroxyphenylacetic acids:11.33  Hydroxyphenylpropanoic acids:6.00 | | 134.94 |  | Phenolic acids | | | Hydroxycinnamic acids:10.89 | | | 10.89 |
|  | Other polyphenols | | | | | Tyrosols:199.23  Other polyphenols:1.82 | | 201.05 |  | Stilbene | | | Stilbenes:0.29 | | | 0.29 |
|  | **Polyphenols total:336.55** | | | | | | | |  | **Polyphenols total:17.45** | | | | | | |
| Olive, black, raw | Flavonoids | | | | | Anthocynins:82.97  Flavones:27.43  Flavonols:49.43 | | 159.83 | kiwi | Flavonoids | Flavanols:0.7 | | | | | 0.7 |
|  |  |  |  |  |  |  |  |  |  | **Polyphenols total:0.7** | | | | | | |
|  | Phenolic acids | | | | | Hydroxybenzoic acids:42.07  Hydroxycinnamic acids:96.42  Hydroxyphenylacetic acids:2.29  Hydroxyphenylpropanoic acids:2.8 | | 143.58 | Grapefruit | Phenolic acids | Hydroxybenzoic acids:14  Hydroxycinnamic acids:7.00 | | | | | 21 |
|  | Other polyphenols | | | | | Hydroxybenzaldehydes:0.1  Tyrosols:265.74 | | 265.84 |  | **Polyphenols total**:21 | | | | | | |
|  |  |  |  |  |  |  |  |  | Orange blond | Flavonoids | Flavanones:44.82  Flavonols:0.10 | | | | | 44.92 |
|  | **Polyphenols total:569.25** | | | | | | | |  | Lignans | Lignans:16.35 | | | | | 16.35 |
|  |  |  |  |  |  |  |  |  |  | **Polyphenols total :61.27** | | | | | | |
| **High calorie** | | | | | | | | | Orange blood | ----- | | | | | | |
| Chocolate dark | Flavonoids | | | | | Flavanols:212.36  Flavonols:25 | | 237.36 | persimmon | Flavonoids | Flavanols:2.27 | | | | | 2.27 |
|  | Phenolic acids | | | | | Hydroxycinnamic acids:24 | | 24 |  | **Polyphenols total:2.27** | | | | | | |
|  | Stilbene | | | | | Stilbenes:0.14 | | 0.14 |  |  |  |  |  |  |  |  |
|  | **Polyphenols total :261.5** | | | | | | | |  |  |  |  |  |  |  |  |
| Chocolate milk | flavonoids | | | | | Flavanols:19.22 | | 19.22 | tangerine | Lignans | Lignans:8.85 | | | | | 8.85 |
|  | **Polyphenols total:19.22** | | | | | | | |  | **Polyphenols total:8.85** | | | | | | |
| **Beverages** | | | | | | | | | Pomegranate | Flavonoids | Flavanols:.1.1 | | | | | 1.1 |
|  |  |  |  |  |  |  |  |  |  | **Polyphenols total:1.1** | | | | | | |
| Black tea | Flavonoids | | | | | Flavanols:74.28  Flavonols:19.3 | | 93.58 | Dried date | Phenolic acids | Hydroxybenzoic acids:16.69  Hydroxycinnamic acids:23 | | | | | 39.69 |
|  | Phenolic acids | | | | | Hydroxybenzoic acids:16.2  Hydroxycinnamic acids:2.61 | | 18.81 |  |  |  |  |  |  |  |  |
|  | **Polyphenols total:112.39** | | | | | | | |  | **Polyphenols total:39.69** | | | | | | |
| Green tea | Flavonoids | | | | | Flavanols:71.18  Flavonols:5.29 | | 76.47 | Fresh date | Phenolic acids | Hydroxybenzoic acids:6.8  Hydroxycinnamic acids:16.38 | | | | | 23.18 |
|  | Phenolic acids | | | | | Hydroxybenzoic acids:9.9  Hydroxycinnamic acids:2.63 | | 12.53 |  |  |  |  |  |  |  |  |
|  | **Polyphenols total:89** | | | | | | | |  | **Polyphenols total:23.18** | | | | | | |
| Oolang tea | Flavonoids | | | | | Flavanols:43.61 | | 43.61 | Fresh plum | Flavonoids | | Anthocynins:47.79  Flavanols:46.90  Flavonols:6.71 | | | | 101.4 |
|  | Phenolic acids | | | | | Hydroxybenzoic acids:5.02 | | 5.02 |  | Phenolic acids | | Hydroxycinnamic acids:89.07 | | | | 89.07 |
|  | **Polyphenols total:48.63** | | | | | | | |  | **Polyphenols total:190.47** | | | | | | |
| Chocolate milk, beverage | Flavonoids | | | | | Flavanols:20.33 | | 20.33 | Strawberry | flavonoids | | Anthocynins:73.01  Flavanols:16.63  Flavonols:2.32 | | | | 91.96 |
|  | Phenolic acids | | | | | Hydroxycinnamic acids:0.9 | | 0.9 |  | Phenolic acids | | Hydroxybenzoic acids:5.67  Hydroxycinnamic acids:7.07 | | | | 12.74 |
|  | **Polyphenols total:21.23** | | | | | | | |  | Stilbenes | | Stilbenes:0.35 | | | | 0.35 |
|  |  |  |  |  |  |  |  |  |  | **Polyphenols total:105.05** | | | | | | |
| Coffee beverage, filter | Phenolic acids | | | | | Hydroxycinnamic acids:212.15 | | 212.15 | banana | Flavonoids | | Flavanols:3.69 | | | | 3.69 |
|  | Other polyphenols | | | | | Alkymethoxyphenols:1.1  Alkphenols:0.27  Methoxyphenols:0.16  Other polyphenols:1.07 | | 2.6 |  | Phenolic acids | | Hydroxybenzoic acids:1.00 | | | | 1.00 |
|  | **Polyphenols total:214.75** | | | | | | | |  | **Total:4.69** | | | | | | |
| Arabica Coffee beverage | Phenolic acids | | | | | Hydroxycinnamic acids:87.71 | | 87.71 | lemon | Flavonoids | | | Flavanones:35.25  Flavones:1.27  Flavonols :0.37 | | | 36.89 |
|  | Other polyphenols | | | | | Alkphenols:0.28  Other polyphenols:0.93 | | 1.21 |  | Lignans | | | Lignans:0.02 | | | 0.02 |
|  | **Polyphenols total:88.92** | | | | | | | |  | **Polyphenols total:36.91** | | | | | | |
| Robusta Coffee beverage | Phenolic acids | | | | | Hydroxycinnamic acids:204.61 | | 204.61 | Lime | Flavonoids | | | Flavanones:46.40  Flavonols :0.40 | | |  |
|  | **Polyphenols total:204.61** | | | | | | | |  | **Polyphenols total:46.80** | | | | | | |
| Coffee beverage, decaffeinated | Phenolic acids | | | | | Hydroxycinnamic acids:278.51 | | 278.51 | Grape , raisin | Flavonoids | | | Flavonols:0.51 | | | 0.51 |
|  | Other polyphenols | | | | | Alkymethoxyphenols:1.93  Methoxyphenols:0.27  Other polyphenols:0.11 | | 2.31 |  | Phenolic acids | | | Hydroxybenzoic acids:0.32  Hydroxycinnamic acids:12.83 | | | 13.15 |
|  | **Polyphenols total:280.82** | | | | | | | |  | **Polyphenols total:13.66** | | | | | | |
| **Jams** | | | | | | | | | Apple juice, pure | **Polyphenols total:35.80** | | | | | |  |
| Plum jam | **Polyphenols total:142.65** | | | | | | |  | Apple juice, concentrate | Flavonoids | | Dihydrochalcones:1.24  Flavanols:7.69  Flavonols:0.09 | | | | 9.02 |
|  |  |  |  |  |  |  |  |  |  | Phenolic acids | | Hydroxycinnamic acids:4.33 | | | | 4.33 |
|  |  |  |  |  |  |  |  |  |  | **Polyphenols total:13.35** | | | | | | |
| Quince jam | Flavonoids | | | | | | Flavanols:7.13  Flavonols:0.83 | 7.96 | Orange (blond) juice , pure | Flavonoids | | Flavanones:37.63  Flavones:13.82  Flavonols:1.08 | | | | 52.53 |
|  | Phenolic acids | | | | | | Hydroxybenzoic acids:0.67  Hydroxycinnamic acids:5.7 | 6.37 |  | Other polyphenols: | | Other polyphenols:2.22 | | | | 2.22 |
|  | Other polyphenols: | | | | | | Other polyphenols:2.69 | 2.69 |  | **Polyphenols total:54.75** | | | | | | |
|  | **Polyphenols total:17.02** | | | | | | | |  |  |  |  |  |  |  |  |
| Raspberry jam | Flavonoids | | | | | | Anthocynins:2.08  Flavonols:3.34 | 5.42 | Orange(blood)juice , pure | Flavonoids | | Anthocynins:3.17  Flavanones:50.84  Flavones:0.61  Flavonols:0.08 | | | | 54.7 |
|  | Phenolic acids | | | | | | Hydroxybenzoic acids:2.35 | 2.35 |  | Phenolic acids | | Hydroxycinnamic acids:1.52 | | | | 1.52 |
|  | **Polyphenols total:7.77** | | | | | | | |  | **Polyphenols total:56.22** | | | | | | |
| Apple jam | Flavonoids | | | | | | Dihydrochalcones:0.4 | 0.4 | Orange(blond)juice, concentrate | Flavonoids | | Flavanones:61.3  Flavones:4.01  Flavonols:0.07 | | | | 65.38 |
|  | **Polyphenols total:0.4** | | | | | | | |  | **Polyphenols total:65.38** | | | | | | |
| Apricot jam | Flavonoids | | | | | | Flavanols:0.97  Flavonols:1.66 | 2.63 | Orange(blood)juice, concentrate | Flavonoids | | Flavanones:60.93  Flavones:0.77  Flavonols:0.03 | | | | 61.73 |
|  | Phenolic acids | | | | | | Hydroxycinnamic acids:3.97 | 3.97 |  | **Polyphenols total:61.73** | | | | | | |
|  | **Polyphenols total:6.6** | | | | | | | |  |  |  |  |  |  |  |  |
| Strawberry jam | Flavonoids | | | | | | Anthocynins:1.23  Flavonols:0.9 | 1.32 | Cantaloupe juice | No data | | | | | |  |
|  | **Polyphenols total:1.32** | | | | | | | |  |  |  |  |  |  |  |  |
|  |  | | | | | | |  | Berries and dried berries  Green plum | No data | | | | | |  |
|  |  |  |  |  |  |  |  |  | Apple ,compote | Flavonoids | | | | | Dihydrochalcone:1.42 | 1.42 |
|  |  |  |  |  |  |  |  |  |  | **Polyphenols total:1.42** | | | | | | |
| **Legumes** | | | | | | | | | **Cereals and cereal products** | | | | | | | |
| Lentil | Flavonoids | | | | | Flavanols:5.17  Flavones :0.95  Flavonols:1.07 | | 7.19 | Bread refined flour | Lignans | | | Lignans:8.48 | | | 8.48 |
|  | Phenolic acids | | | | | Hydroxybenzoic acids:0.46  Hydroxycinnamic acids:2.63 | | 3.09 |  | **Polyphenols total:8.48** | | | | | | |
|  | Stilbenes | | | | | Stilbenes:0.09 | | 0.09 |  |  |  |  |  |  |  |  |
|  | **Polyphenols total:10.37** | | | | | | | |  |  |  |  |  |  |  |  |
| Common bean, whole, raw | Flavonoids | | | | | Flavanols:1.12  Flavonols: 49.96  Isoflavonoids: 0.50 | | 51.58 | Bread common wheat, whole grain | Other polyphenols | | | Alkymethoxyphenols:24.71 | | | 24.71 |
|  | **Polyphenols total::51.58** | | | | | | | |  | **Polyphenols total:24.71** | | | | | | |
| Common bean, dehulled, raw | Phenolic acids | | | | | Hydroxycinnamic acids:0.53 | | 0.53 | Bread , rye whole grain | Phenolic acids | | | Hydroxybenzoic acids:0.57  Hydroxycinnamic acids:4.67 | | | 5.24 |
|  | **Polyphenols total:0.53** | | | | | | | |  | Other polyphenols | | | Alkymethoxyphenols:61.94 | | | 61.94 |
|  |  |  |  |  |  |  |  |  |  | **Polyphenols total:67.18** | | | | | | |
| Mung bean | Lignans | | | | | Lignans:0.18 | | 0.18 | Pasta | Other polyphenols | | | Alkymethoxyphenols:4.83 | | | 4.83 |
|  | **Polyphenols total:0.18** | | | | | | | |  |  | | | | | | |
| Broad bean, whole, raw | Flavonoids | | | | | Flavanols:49.37 | | 49.37 | Pasta,whole grain | Other polyphenols | | | Alkymethoxyphenols:21.85 | | | 21.85 |
|  | **Polyphenols total:49.37** | | | | | | | |  | **Polyphenols total:21.85** | | | | | | |
| Broad bean, dehulled, raw | Phenolic acids | | | | | Hydroxycinnamic acids:3.1 | | 3.1 | Rice , refined | Phenolic acids | | | Hydroxybenzoic acids:0.28  Hydroxycinnamic acids:0.34 | | | 0.62 |
|  | **Polyphenols total:3.1** | | | | | | | |  | **Polyphenols total:0.62** | | | | | | |
| Chickpea whole, raw | Lignans | | | | | Lignans:2.00 | | 2.00 | Rice, parboiled | Phenolic acids | | | Hydroxybenzoic acids:0.38  Hydroxycinnamic acids:0.70 | | | 1.08 |
|  | **Polyphenols total:2.00** | | | | | | | |  | **Polyphenols total:1.08** | | | | | | |
| Chickpea dehulled, raw | Phenolic acids | | | | | Hydroxybenzoic acids:5.20  Hydroxycinnamic acids:4.30 | | 9.50 | Rice, whole grain | Phenolic acids | | | Hydroxybenzoic acids:0.33  Hydroxycinnamic acids:0.17 | | | 0.5 |
|  | **Polyphenols total:9.50** | | | | | | | |  | **Polyphenols total:0.5** | | | | | | |
| Soy bean, roasted | Flavonoids | | | | | Isoflavonoids:246.94 | | 246.94 | Baked potato | **Polyphenols total:0** | | | | | |  |
|  | **Polyphenols total:** 246.94 | | | | | | | |  |  |  |  |  |  |  |  |
| Cotyledons | No data | | | | | | |  | French fries | No data | | | | | |  |
| **Meats** | | | | | | | | | Common wheat, germ | Phenolic acids | | Hydroxybenzoic acids:3.65  Hydroxycinnamic acids:163.24 | | | | 166.89 |
|  |  |  |  |  |  |  |  |  |  | Lignans | | Lignans:9.02 | | | | 9.02 |
|  |  |  |  |  |  |  |  |  |  | **Polyphenols total:175.91** | | | | | | |
| Soy meat | Flavonoids | | | | | Isoflavonoids:74.59 | | 74.59 | Common wheat, whole grain | Flavonoids | | Flavones:77.29 | | | | 77.29 |
|  |  |  |  |  |  |  |  |  |  | Phenolic acids | | Hydroxycinnamic acids:0.23 | | | | 0.23 |
|  |  |  |  |  |  |  |  |  |  | Other polyphenols | | Alkylphenols: 64.14 | | | | 64.14 |
|  | **Polyphenols total:** 74.59 | | | | | | | |  | **Polyphenols total:141.66** | | | | | | |
| Soy, sausage | flavonoids | | | | | Isoflavonoids:27.76 | | 27.76 | Common wheat, refined flour | Flavonoids | | Flavones:18.4 | | | | 18.4 |
|  |  |  |  |  |  |  |  |  |  | Phenolic acids | | Hydroxybenzoic acids:0.09  Hydroxycinnamic acids:8.18 | | | | 8.27 |
|  |  |  |  |  |  |  |  |  |  | Other polyphenols | | Alkylphenols: 1.89 | | | | 1.89 |
|  | **Polyphenols total:27.76** | | | | | | | |  | **Polyphenols total:28.56** | | | | | | |
| **Milk** | | | | | | | | |  |  | | | | | |  |
| Chocolate milk | Flavonoids | | | | | Flavanols:19.22 | | 19.22 |  |  | | | | | |  |
|  | **Polyphenols total:19.22** | | | | | | | |  |  |  |  |  |  |  |  |
| **Vegetables** | | | | | | | | | Vermicelli, noodles | No data | | | | | |  |
| Lettuce | Flavonoids | | | | | Flavones:0.4  Flavonols :3.89 | | 3.93 | Barley, whole grain flour | Flavonoids | | | Flavanols:35.2 | | | 35.2 |
|  | Phenolic acids | | | | | Hydroxycinnamic acids:3.78 | | 3.78 |  | Other polyphenols | | | Alkylphenols: 4.51 | | | 4.51 |
|  | **Polyphenols total:7.71** | | | | | | | |  | **Polyphenols total:39.71** | | | | | | |
| Tomato | Flavonoids | | | | | Flavanones:0.14  Flavonols:5.37 | | 5.51 | Sorghum , whole grain | Phenolic acids | | | Hydroxybenzoic acids:2.55 | | | 2.55 |
|  | Phenolic acids | | | | | Hydroxycinnamic acids:3.86 | | 3.86 |  | **Polyphenols total:2.55** | | | | | | |
|  | **Polyphenols total:9.37** | | | | | | | |  |  |  |  |  |  |  |  |
| Tomato cherry | Flavonoids | | | | | Flavonols :14.76 | | 14.76 |  |  | | | | | |  |
|  | Phenolic acids | | | | | Hydroxycinnamic acids:3.74 | | 3.74 |  |  |  |  |  |  |  |  |
|  | **Polyphenols total:18.5** | | | | | | | |  |  |  |  |  |  |  |  |
| Cucumber | Flavonoids | | | | | Flavones:6.5  Flavonols :0.1 | | 6.6 |  |  | | | | | |  |
|  | Lignans | | | | | Lignans:8.06 | | 8.06 |  |  |  |  |  |  |  |  |
|  | **Polyphenols total:14.66** | | | | | | | |  |  |  |  |  |  |  |  |
| Eating vegetables | **Polyphenols total:60.99** | | | | | | |  | **Spices** | | | | | | | |
|  |  | | | | | | |  | Pepper spice, green | **Polyphenols total:380** | | | | | | |
| Squash | Lignans | | | | | Lignans:9 | | 9 | Pepper spice , black | **Polyphenols total:1000** | | | | | |  |
|  | **Polyphenols total:9** | | | | | | | |  |  |  |  |  |  |  |  |
| Zucchini | Flavonoids | | | | | Flavonols:1.32 | | 1.32 | Turmeric | Other polyphenols | | | | Curcuminoids:  5433.57 | | 5433.57 |
|  | **Polyphenols total:1.32** | | | | | | | |  | **Polyphenols total:5433.57** | | | | | | |
| Pumpkin | Flavonoids | | | | | Flavones:1.63 | | 1.63 |  |  | | | | | |  |
|  | Lignans | | | | | Lignans:6.61 | | 6.61 |  |  |  |  |  |  |  |  |
|  | **Polyphenols total:8.24** | | | | | | | |  |  |  |  |  |  |  |  |
| Baked Eggplant | No data | | | | | | |  |  |  | | | | | |  |
| Celery stalk | Other polyphenols | | | | | Furanocoumarins:2.59 | | 2.59 |  |  | | | | | |  |
|  | **Polyphenols total:2.59** | | | | | | | |  |  |  |  |  |  |  |  |
| Celery leaves | Flavonoids | | | | | Flavones:133.38 | | 133.38 |  |  | | | | | |  |
|  | **Polyphenols total:133.38** | | | | | | | |  |  |  |  |  |  |  |  |
| Green pea | No data | | | | | | |  |  |  | | | | | |  |
| Green bean | Flavonoids | | | | | Flavanols:2.42  Flavonols: 5.55 | | 7.97 |  |  | | | | | |  |
|  | **Polyphenols total:7.97** | | | | | | | |  |  |  |  |  |  |  |  |
| Carrot, raw | Phenolic acids | | | | | Hydroxybenzoic acids:0.05  Hydroxycinnamic acids:18.21 | | 18.26 |  |  | | | | | |  |
|  | **Polyphenols total:18.26** | | | | | | | |  |  |  |  |  |  |  |  |
| Cooked carrot | No data | | | | | | |  |  |  | | | | | |  |
| Garlic | Lignans | | | | | Lignans:2.97 | | 2.97 |  |  | | | | | |  |
|  | **Polyphenols total:2.97** | | | | | | | |  |  |  |  |  |  |  |  |
| White onion | Flavonoids | | | | | Flavonols:5.4 | | 5.4 |  |  | | | | | |  |
|  | **Polyphenols total:5.4** | | | | | | | |  |  |  |  |  |  |  |  |
| Red onion | Flavonoids | | | | | Anthocynins:9.00  Flavanols:128.51 | | 137.51 |  |  | | | | | |  |
|  | Phenolic acids | | | | | Hydroxybenzoic acids:2.00 | | 2.00 |  |  |  |  |  |  |  |  |
|  | **Polyphenols total:139.51** | | | | | | | |  |  |  |  |  |  |  |  |
| Welsh onion | Flavonoids | | | | | Flavanones:7.02  Flavones:14.00  Flavonols: 12.00 | | 33.02 |  |  | | | | | |  |
|  | Phenolic acids | | | | | Hydroxycinnamic acids:0.02 | | 0.02 |  |  |  |  |  |  |  |  |
|  | **Polyphenols total:33.04** | | | | | | | |  |  |  |  |  |  |  |  |
| Fried onion | No data | | | | | | |  |  |  | | | | | |  |
| Red cabbage | Flavonoids | | | | | Flavones:0.2  Flavonols :0.43 | | 0.63 |  |  | | | | | |  |
|  | Lignans | | | | | Lignans:11.09 | | 11.09 |  |  |  |  |  |  |  |  |
|  | **Polyphenols total:11.72** | | | | | | | |  |  |  |  |  |  |  |  |
| White cabbage | Flavonoids | | | | | Flavones:0.1  Flavonols :0.92 | | 1.02 |  |  | | | | | |  |
|  | Lignans | | | | | Lignans:10.03 | | 10.03 |  |  |  |  |  |  |  |  |
|  | **Polyphenols total:11.05** | | | | | | | |  |  |  |  |  |  |  |  |
| Green cabbage | Flavonoids | | | | | Flavonols :0.04 | | 0.04 |  |  | | | | | |  |
|  | Lignans | | | | | Lignans:12.77 | | 12.77 |  |  |  |  |  |  |  |  |
|  | **Polyphenols total:12.81** | | | | | | | |  |  |  |  |  |  |  |  |
| Purple cabbage | flavonoids | | | | | Flavones:1.1  Flavonols :0.02 | | 1.12 |  |  | | | | | |  |
|  | **Polyphenols total:1.12** | | | | | | | |  |  |  |  |  |  |  |  |
|  |  | | | | | | |  |  |  | | | | | |  |
| Allspice | No data | | | | | | |  |  |  | | | | | |  |
| Baked spinach | No data | | | | | | |  |  |  | | | | | |  |
| Chilli pepper | flavonoids | | | | | Flavones:2.88  Flavonols :9.93 | | 12.81 |  |  | | | | | |  |
|  | **Polyphenols total:12.81** | | | | | | | |  |  |  |  |  |  |  |  |
| Sweet pepper, green | Flavonoids | | | | | Flavones:2.11  Flavonols: 2.16 | | 4.27 |  |  | | | | | |  |
|  | Phenolic acids | | | | | Hydroxycinnamic acids:0.45 | | 0.45 |  |  |  |  |  |  |  |  |
|  | **Polyphenols total:4.72** | | | | | | | |  |  |  |  |  |  |  |  |
| Spinach, raw | Flavonoids | | | | | Flavonols :119.27 | | 119.27 |  |  | | | | | |  |
|  | **Polyphenols total:119.27** | | | | | | | |  |  |  |  |  |  |  |  |
| Turnip white, raw | **Polyphenols total:0** | | | | | | |  |  |  | | | | | |  |
| Turnip, root | Lignans | | | | | Lignans:17.96 | | 17.96 |  |  | | | | | |  |
|  | **Polyphenols total:17.96** | | | | | | | |  |  |  |  |  |  |  |  |
